# Capturing the Role of Context in Complex System Change: An Application of the Canadian Context and Capabilities for Integrating Care (CCIC) Framework to an Integrated Care Organisation in the UK

**DOI:** 10.5334/ijic.5196

**Published:** 2020-02-11

**Authors:** Sheena Asthana, Felix Gradinger, Julian Elston, Susan Martin, Richard Byng

**Affiliations:** 1 University of Plymouth, UK; 2 Torbay and South Devon NHS Foundation Trust, UK

**Keywords:** integrated care, complex intervention, context, CCIC framework

## Abstract

**Introduction:**

If integrated care approaches are to be properly adapted to local contexts, a better understanding is required of key determinants of implementation and how these might be appropriately supported.

**Purpose:**

This study applied the Canadian Context and Capabilities for Integrating Care (CCIC) Framework to investigate factors influencing the implementation and outcomes of a complex integrated care change programme in Torbay and South Devon (TSD) and, more specifically, in one of five sub-localities, Coastal.

**Methods:**

A case study method using embedded ‘Researchers in Residence’ to conduct action-based participatory research and deploying mixed qualitative methods.

**Results:**

The relative importance of some domains differ between the English and Canadian studies. In this case study, physical features (structural and geographic) were found to be very pertinent to the relative success of the Coastal Locality, as were empowered clinical leadership, with readiness for change being expressed through processes and cultures that were risk-enabling, strengths-based, person-/outcome-focused.

**Conclusions:**

The CCIC Framework provided a useful tool capturing key elements of complex system change with key domains being transferable across settings, while also finding local variation in the UK. This would encourage its wider application so that further comparisons can be made of the ways in which different contextual and implementation properties impact upon delivery and outcomes.

## Background

### Introduction

Health care systems worldwide are striving for the ‘triple aim’ of better health for their populations, improved experience of care for patients and lower system costs [1]. In England, as elsewhere, care integration is seen as the key to improving services for patients and unlocking efficiencies [2,3]. The recent NHS Long-Term Plan [4] promotes new organisational forms and service delivery models, with greater levels of horizontal and vertical integration, emphasising better inter-professional working, more flexibility of roles and closer working between primary, community and secondary care services as well as with the voluntary sector to promote a ‘person-centred approach’ [5]. It is assumed that this strategy provides a means of addressing high levels of demand on acute care from an ageing population, often with multiple, long-term and complex conditions [4,6,7,8].

The Long-Term Plan is the latest of a series of policy initiatives that have been launched to support service integration in England [2,9,10,11]. Thus, there has been a significant policy investment. Yet, progress with national integration initiatives has been more limited than hoped for [12]. Moreover, the existing evidence base in England does not yet support the proposition that integration saves money, reduces hospital activity or improves patient outcomes [13]. Several factors are at work, including the difficulties of achieving innovation in a challenging financial context [8] and the time required to embed new ways of working. For example, early evaluation of the Integrated Care and Support Pioneers Programme found that local areas expected that it would take five years or more to produce demonstrable impacts [14]. Most importantly (and the focus of this paper), a very complex array of factors can influence the adoption, non-adoption, abandonment, spread, scale up and sustainability of innovation in service organisations [15,16,17,18,19,20]. Capturing this complexity is central to enabling a better understanding of key determinants of implementation success or failure and facilitating the development of integrated care approaches that are properly adapted to local contexts [21,22,23,24].

### Theoretical frameworks for the evaluation and implementation of integrated care

Many models and frameworks have been published with a view to improving and evaluating care integration. Updating Van Houdt et al. (2013) [25], a recent review identified 35 frameworks explicitly described as being developed for care coordination [26,27]. These vary in conceptualisation, design and complexity; serve different purposes (e.g. to describe/guide the implementation process, to identify determinants of implementation or to evaluate implementation); focus on different levels (e.g. from intervention to the organisation to the whole-system); and differ in their degree of operationalizability, i.e. the extent to which methodological tools are clearly stated to promote consistent application [28]. They also draw on different antecedents [28,29,30,31,32,33,34].

Most frameworks identify a similar range of factors as determinants of implementation success or failure [20]. Our decision to apply the Context and Capabilities for Integrating Care (CCIC) Framework [30,31] to an evaluation of integrated care in Torbay and South Devon NHS Foundation Trust, an integrated care organisation, was informed by several factors, the first being a sensitivity to the importance of *context* in the design and evaluation of complex interventions and the assessment of what works for whom in what circumstances [35,36]. We concur with the definition of context as “a set of characteristics and circumstances that consist of active and unique factors, within which the implementation is embedded. As such, context is not a backdrop for implementation, but interacts, influences, modifies and facilitates or constrains the intervention and its implementation … It is an overarching concept, comprising not only a physical location but also roles, interactions and relationships at multiple levels” [37, p. 8]. This complex and versatile definition is not easily accommodated in models that, for example, distinguish between the inner and the outer setting [34] or micro-, meso- and macro scales [29]. We wanted a framework that better captured the modifying role of local contextual factors.

The context and implementation of complex interventions (CICI) framework [37] might have offered a solution. This framework excels in identifying the active and unique characteristics (geographical, epidemiological, sociocultural, socioeconomic, ethical, legal and political) that modify, facilitate or constrain intervention delivery and effects. Yet, taken to its extreme, the argument that every context is unique might imply that interventions cannot be more widely replicated. This is difficult to reconcile with a policy context that is seeking to learn lessons from and transfer generalised interventions that have a theoretical, logical and empirical basis. Moreover, variation in effects may arise from other differences in system factors. The complex interventions framework [37] neatly summarises these as implementation process, strategies and agents. This may not be sufficiently nuanced nor acknowledge the practical reality of *what* complex system change can reasonably target and achieve. A framework that was less narrowly focused but that incorporated a more manageable (i.e. less detailed) degree of the comprehensiveness of models such as the Consolidated Framework for Implementation Research [33] and the EU-funded SELFIE [29] was required.

In a field where transferring ‘evidence-informed’ interventions to different contexts is important, it would be helpful to *compare* how different contextual and implementation properties impact upon delivery and outcomes in a way that usefully informs how interventions might be usefully adapted and which contextual factors add to the level of uncertainty in transferability.

The Canadian CCIC Framework [30,31] addressed these concerns. Within this model, context is framed as dynamic organisational capabilities. Many of these are both structural and process-oriented in nature (e.g. governance, leadership, information technology and partnership working). There is a recognition of the difficulties of demarking boundaries, factors such as information technology, inter-professional teamwork and partnerships relating to both organizational capabilities and the intervention itself. The model is comprehensive, but not overly so. It also, importantly, offers the opportunity to identify, validate (and thus compare) factors that are ranked as most important to care integration, providing us with some baseline against which to assess readiness to integrate [27].

## Research objectives

Against this background, the main objective of this study was to use the Canadian CCIC framework to investigate factors influencing the implementation and outcomes of a complex integrated care change programme in Torbay and South Devon (TSD) and, more specifically, in one of five sub-localities, the performance of which is significantly higher than that of its neighbours (as reported more quantitatively [38] elsewhere). The secondary objectives were to (a) explore the extent to which critical factors in the CCIC framework [30] were also pertinent in the UK context and (b) identify key contextual and intervention properties that can be feasibly adapted to maximise the capacity of organizations to implement integrated care.

## Methods

### Setting

TSD is a coastal and moorland area in South West England, covering some 907 square kilometres, including 121 kilometres of coastline. It has a population of approximately 293,406 (rising by around 100,000 during the summer months). TSD ICO provides acute and community services organised around five localities, which range in size from 36,251 to 72,692 registered residents. It was one of the first ICOs in England, formed out of a merger between the acute NHS Trust and Torbay Care Trust in October 2015, a provider of adult social care and community services. The area has a strong history of horizontal service integration, following the creation of the Torbay Care Trust in 2005, with jointly commissioned and managed health and social care teams, pooled budgets and a risk sharing agreement between the Trust and the Torbay Council social services [39,40].

From March 2017, several changes were made to the organisation and delivery of care in the ICO to promote responsive and dependable out-of-hospital services that provide high quality assessment and support to people in their own homes. Five of the nine community hospitals in four localities (the largest, Torquay, houses an acute hospital) were closed, resulting in a reduction in the number of community hospital beds from 159 to 93. In their place, intermediate care teams (comprising nurses and therapists) were expanded to include GPs and more recently pharmacists; voluntary sector ‘Well-Being Coordinators’ (WBCs) who support social prescribing were appointed; and ‘Health and Well-Being Centres’ established, some in former hospital sites. These comprise community nurses, allied health professionals, social care services and a voluntary team who, in addition to running their core community services, meet regularly as Multi-Disciplinary Teams (MDTs, e.g. around Enhanced Intermediate Care (EIC)). The aim of this new ‘Care Model’ is to focus on well-being and early intervention to keep people well for longer and less likely to escalate in their need for intensive NHS and social care support.

The Coastal locality of interest for this study covers the towns of Teignmouth and Dawlish and their surrounding areas. It has a high proportion of older people compared to England (22% of the 36,251 Coastal locality residents are over 70 years). Despite developing the same Care Model, performance (as measured quantitatively by higher EIC referral rates, lower lengths of episodes for intermediate care, higher rates of home-base care and lower GP referrals to Accident and Emergency (A&E)) is better in Coastal compared with the other localities, with differences that reach statistical significance [38]. The fact that Coastal was an earlier adopter of the new Care Model than other localities may in part explain its better performance. We also hypothesize that local context – and its interactions with interventions – plays a critical role.

### Research design and methods

We applied a responsive evaluation approach to a best practice case study looking at the system, team and individual clients [41,42,43]. This case study therefore offers an exploration of the particular and the personal narrative, combining questions around ‘what works’ with ‘how things work’ and ‘what is going on’. To support this research method, we embedded Researchers in Residence [44,45,46]. At the time of writing, they have been in place for three years, working within the Trust’s Quality Improvement Team and spending significant amounts of time co-located with the Health & Wellbeing Team in Coastal. While adopting multiple methods, an action-based participatory approach lies at the heart of the method, findings being co-produced with stakeholders and members of the public iteratively over the period of immersion. The overall research question focused on the implementation and impact of integrated care model innovations (reported elsewhere) and the delivery of person-centred, coordinated services.

Capturing the research efforts of RiRs who are immersed (daily) in intervention contexts is challenging, not least because many of the interactions between RiRs and stakeholders take place in informal, serendipitous ways operating in the middle ground between quality improvement, service development and evaluation, and implementation research. Across the immersion period and the wider system, informal observation notes were collected from dedicated meetings (n = 112) or naturally occurring events (n = 68). These were based on the principle of including as many staff and stakeholders to represent vertical and horizontal perspectives on integration, to maximise the authenticity of observed dynamics and to minimise the additional burden of further data collection events.

Observed events in the Coastal locality included nine daily Enhanced Intermediate Care MDT Meetings; eight Locality Steering Group Meetings (chaired by the GP/ICO Locality Clinical Director, attended by all community team leads); eight locality engagement group meetings (chaired by community members and facilitated by the Clinical Commissioning Group); four voluntary sector triage/referral meetings; four co-design workshops including practitioners and members of the public; two Volunteering in Health (ViH) team meetings; three in-depth semi-structured interviews with ViH staff (manager and wellbeing coordinators who sit on daily hub MDTs), and one social care team focus group (co-located with wider community team and jointly managed between local government and ICO).

Qualitative data from the action research were recorded with audio and field notes. The reflective and largely unstructured field notes were recorded independently by both RiRs, with the overall research question around implementation and impact in mind. Notes were typically made during or following meetings, written up in Word and stored on a secure, password-protected server. The transcripts were uploaded into an NVIVO file, where they were merged for sub-group analysis relevant to Coastal locality with other documentary materials (official meeting notes, and other documents).

For this analysis, the NVIVO file depository was used for data retrieval (key word searches) to deductively populate the CCIC framework domains with our data and inductive insights. In a succession of iterative encounters between the two RiRs and the locality clinical director, we reflected upon the most salient factors influencing implementation and presented these findings in an interactive workshop at the International Conference on Integrated Care [47]. Subsequently, and with the support of other co-authors an Excel file was populated using CCIC definitions, if/then statements applied to our local context, and vignettes from the data. The results presented below are an aggregation and abstraction of this process, largely abstaining from referencing data points due to space limitations. The intensive engagement of the research team inherently risks self-justificatory bias in reporting activities and outcomes. To minimise that risk, we included ‘clean room’ data analysis by having the data co-analysed by (and this paper co-written with) team members (SA, RB) not personally involved in the practical interventions).

Individual and organisational consent was obtained for the research, while ethical approval was granted through ‘Proportionate Review’ by the NHS Health Research Authority.

## Results

### Critical factors to implementation success or failure

The CCIC framework describes how 17 organisational context and capabilities interact to varying degrees to influence the implementation and outcomes of integrated care interventions. These are grouped into three broad categories – basic structures, people and values, and key processes – which can be examined within an organisation and across a health and social care network or system.

**Figure 1:**
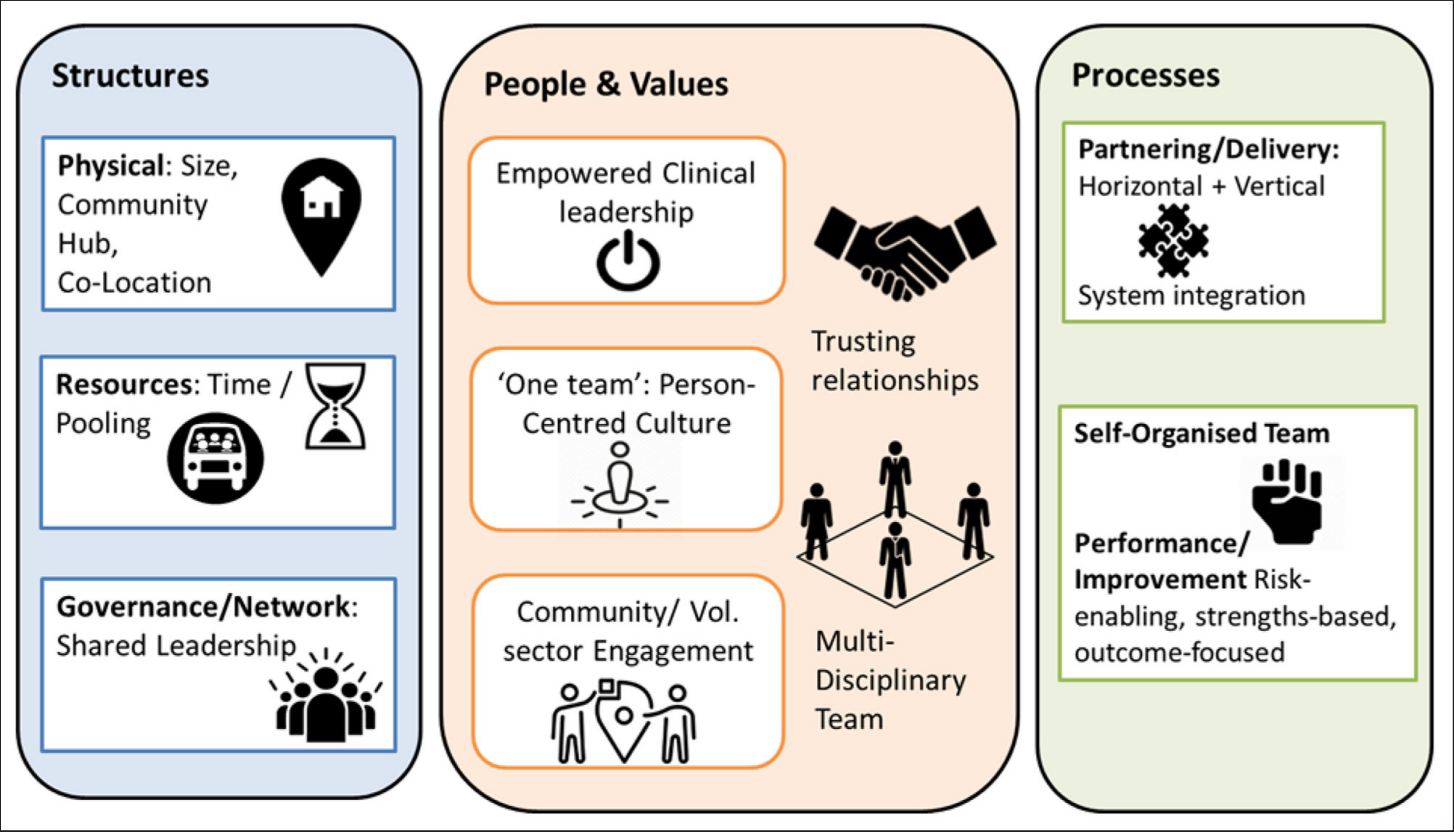
The most salient CCIC domains in the Coastal Locality Case Study.

In the Canadian context [30], nine contextual factors and organisational capabilities were considered more significant than the rest, of which Leadership Approach, Clinical Engagement & Leadership and Readiness for Changes) were the most important of all. In the Coastal Locality case study, we found that shared leadership across the system and empowered clinical leadership in the locality were indeed crucial (Figure 1), with readiness for change being expressed through processes and cultures that were risk-enabling, strengths-based, person-/outcome-focused. However, the relative importance of some domains seemed to differ between the English and Canadian studies.

#### Basic structures

For example, we found *physical features* (structural and geographic characteristics of the organisation/practice and network) to be very pertinent to the relative success of integrated care in the Coastal Locality. This area is sufficiently small (n = 36,251) and, given its composition of coastal *towns*, geographically concentrated enough to allow triage, assessment and referral in one multi-disciplinary team (MDT). In the largest locality, Torquay (72,692 registered residents), the single MDT initially had a caseload of around 60 patients. This proved unmanageable. The MDT has since been reorganised into two teams, which has significantly improved their ability to be person-centred, as well as improve staff morale.

Spatial concentration and the small size of the Coastal Locality have also allowed relationships to develop (supporting readiness for change) over time. There is a history of collaboration (e.g. between GPs and community hospital teams and between health and social care) that predates the new Care Model. Health and social care professionals also have good working relationships with the local private care market (which is too small to make block contracts feasible so intermediate care beds are spot-purchased) and the voluntary sector. A manageable population size perhaps makes it easier to understand population needs (“we know and own our population”) and the roles of other local providers in meeting those needs, nourishing a place-based and tailored approach. It has also made possible the co-location of the Health and Well-Being team (including voluntary sector WBCs) in one building. The two market towns are close enough (5.3 km) to allow community-based services to be delivered from a single hub. Both the hub and community hospital clearly see themselves as one team.

While clinical hubs and locality teams have been established in other localities, there are significant challenges in the more rural parts of the ICO with respect to achieving economies of scale, managing partnerships and understanding a widely dispersed population. Different solutions have been required here, such as the use of video meeting technology to maintain good communication.

Resources are important to implementation success. In the Coastal Locality, inpatient beds have been retained in one community hospital, while the other has been converted into a Health and Well-being Centre in which various teams (including voluntary sector WBCs) are co-located. The Centre also co-locates and has a joint management structure with social care services provided by the local authority. In addition to providing office space for genuine co-location, the building has a designated meeting room for MDT meetings. In some localities, these are held in large busy offices where there are significant opportunities for distractions (such as emails, phone calls and other visitors). Indeed, in some cases, the room is not big enough to accommodate all members of the MDT. These factors have a bearing on the focus and flow of meetings, the quality of interaction and, in turn, decision-making.

Structures of accountability are significant in the Coastal Locality insofar as a strong culture has evolved around *self-organised teams* (“we don’t ask for permission, but forgiveness”). The term is loaded academically but in the context of this case study means that the Coastal team iteratively evolved to have a shared vision and ownership of improvement, empowering itself to work with considerable autonomy in terms of managing inter-professional dynamics, and clinically driven decision-making. Factors that have enabled self-organisation include geography (see above); a history of collaboration; and the presence of ‘early adopter’ professionals who have worked for long enough in the area to have established good working relationships and developed the confidence to champion new ways of working such as pooling resources and blurring roles (e.g. doing joint assessments and having integrated training pathways for social workers).

We found information technology to be less pertinent to understanding why the Coastal Locality had enjoyed relative success. Coastal was initially digitally immature with respect to still relying on paper records (especially in nursing teams). In the absence of connected IT systems, the efficiency and coordination of integrated care deorganisational commitment appears to have been facilitated by clinician-led communication and co-location. Indeed, there were initially mixed feelings about the introduction of shared access to the primary care system (“it feels less personal when not using our own notes”), though this has now bedded down well and an integrated IT solution is now being rolled out.

#### Peoples and values

In the Canadian study, leadership – and particularly clinical leadership and engagement – were identified as critical factors of organisational readiness for integrated care. We found this to be highly significant in the Coastal Locality, in a way that suggests a strong role for *shared, bottom-up leadership* (as opposed to the centralised and standardised roll-out of service innovations). Frontline clinicians in the Coastal Locality were notable for having trusting and friendly relationships between team leads (Physio, Nurses and Matrons, Social Care), general workforce stability with long experience and a critical mass of innovators (“we always say yes”) across professional groups.

The decision by the ICO to appoint GPs as locality clinical directors on employed contracts was pivotal. In Coastal, this has been a key factor, in part because of the GP’s personal skill set (less easily replicable). Engagement with a small group of general practitioner colleagues was also pivotal. Well embedded in their patch and determined to enjoy a high degree of clinical and organisational autonomy while working within an integrated system, this group showed real interest in adapting integrated care interventions to local ways of working. The clinical directors link the GP community directly into the ICO which has contributed to overcoming barriers between the acute and primary sectors, including helping to negotiate contracted GP attendance at MDTs. The involvement of these key players was also characterised by a sustained focus on *patient-centredness & engagement* (reflected in both consultation events and an openness to ongoing reflection and evaluation, systematic assessments of patient and caregiver outcomes and experience acknowledged as key to improving care planning, coordination and transition between services). We conclude that the size of population and local assets allowed clinician-led partnerships to nourish trusting relationships and hold pro-active, holistic knowledge that supported the delivery of person-centred, coordinated care.

Decision-making in the Coastal Locality was also flattened, resulting in more informal and less formal meetings and a strategy to train, value and empower non-medical staff (e.g. through rotational posts). A *commitment to learning* (in terms of engaging with the RiRs’ evaluation of the care model and devoting time to forums of reflection and analytical capacity) was another factor that we would rank as a critical determinant of success in the Coastal Locality. For example, the Coastal leads insisted on collecting their own performance data despite being asked to discontinue doing so and, with RiR input, devised their own performance management system (*measuring performance*). This is now being replicated across TSD.

The broader lessons to draw from this are complex. There is certainly evidence to suggest the benefits of self-organisation. In the Coastal Locality, the development of informal, bottom-up, devolved, clinician-led, co-located and jointly managed locality teams seems to be related to a willingness to engage, own and drive change. This in turn increased job satisfaction (as observed in numerous “Zoo” events when the team welcomed and represented their work to visitors) which would in turn be expected to improve organisational commitment and productivity [48].

#### Key processes

With respect to processes, CCIC identifies partnering and the organisation of inter-professional teamwork and joint care planning as critical determinants of success. We agree that informal partnership is crucial and that this relies on trusting relationships at all levels. Formal governance structures are only enablers to this. In this case study, a balance of top-down and bottom-up committees and system-wide steering functions facilitated the emergence over time of trusting relationships and iteratively evolving teams at locality level. Examples include the bi-monthly Care Model Delivery Group with senior vertical/horizontal representation; monthly locality steering group meetings with all team leads; monthly Clinical Commissioning Group engagement committees with wider public representation; daily MDTs and separate professional team meetings. These system-wide partnerships together with proximity and co-location further embedded vertical and horizontal integration. This in turn impacted upon service delivery (which was facilitated by informal and formal inter-professional working and communication). High re-referral rates to intermediate care and more efficient use of available beds within the locality evidenced by shorter lengths of stay and more interventions at home are indicative or service improvements.

With respect to the processes embedded in the CCIC, we would prioritise measuring performance as a key determinant (a factor not highlighted as critical in the Canadian study). As noted above, there was a willingness in Coastal to genuinely question the process and outcomes of integrated care. Locality-owned governance, accountability and processes that are risk-enabling, strengths-based, outcome-focused and patient-centred appear to have influenced care delivery by allowing a self-organised locality team to take control of tailoring services (as evidenced by observations of role-blurring within and between multi-professional teams, allowing voluntary sector staff to attend MDTs and undertaking joint patient assessments). These developments took place without any specific financial incentives.

A theme here was around the balance between informal accountability where risk-enabling and ownership was shared within the self-organised team and formal accountability (which tends to revolve around key national performance indicators). One of the key features of stakeholders in the Coastal Locality was an understanding that, while the system is ultimately accountable to short, medium and long-term outcomes, logic modelling suggests that benefits are likely to accrue in the longer term, requiring a different approach to evaluation and ultimately service delivery.

## Discussion

As we have reported elsewhere [38], the Coastal Locality outperforms its neighbours in Torbay and South Devon with respect to higher EIC referral rates, lower lengths of episodes for intermediate care, higher rates of home-base care and lower GP referrals to Accident and Emergency. This is despite the fact that these localities belong to the same Integrated Care Organisation which has promoted the same Care Model. In seeking to understand why such variation in performance has arisen, we made the case for prioritising context in our investigation of factors influencing system change; a requirement that we felt was addressed by the Context and Capabilities for Integrating Care (CCIC) Framework [30,31]. The CCIC Framework worked well in this case study, its domains feeling relevant to the real-life experience of key factors influencing the evolution of the Care Model.

Our results confirm the critical importance of context (which we understand to comprise “not only a physical location but also roles, interactions and relationships at multiple levels” [37, p. 8]). For example, leadership in the Coastal locality has been a collective and emergent process, contextually situated rather than something individual and prescribed [49]. Being a well-established clinical workforce in a small, geographically concentrated area has undoubtedly facilitated the development of trusting inter-professional relationships. Sharing a relatively small but complex caseload has also reinforced the importance of cooperation and collaboration. There is a strong sense of a shared vision in the locality. As a result, solution-driven interactions start with needs of the person needing support. Services are tailored to deliver the best outcomes. Such an approach requires professional autonomy and flexibility, a pre-requisite of self-organisation [50].

While this process has not been driven by top-down management, the ICO did develop the broad parameters of its New Care Model and is actively seeking to identify and replicate key ingredients for success. If one understands the Care Model in this locality to have benefitted from the seemingly serendipitous convergence of a range of (mainly contextual) factors, then the organisational capabilities that have developed in Coastal cannot be simply transferred elsewhere. On the other hand, identifying which capabilities are important for integrating care and why does offer the possibility of learning wider lessons for both the ICO and other areas seeking to develop integrated care systems.

For example, we would argue that size matters. The Coastal Locality has undoubtedly benefitted from having a lower caseload than its neighbours, which means that MDT meetings that are attended by a range of professionals and support staff to co-ordinate actions and outcomes that support care and treatment are manageable. There may be important implications for Primary Care Networks (PCNs), a new NHS policy development, the purpose of which is to enable greater provision of proactive, personalised, coordinated and more integrated health and social care [51]. The PCN policy states that networks should serve communities of around 30,000 to 50,000 (small enough to provide the personal care valued by both patients and GPs, but large enough to have impact and economies of scale through better collaboration between practices and others in the local health and social care system). Our research suggests that forming networks at the lower end of the population size envisaged by NHS England may lead to better partnership working and outcomes. Moreover, the Coastal locality will benefit from the fact that its PCN is coterminous with the locality boundaries. In other parts of the ICO, new PCN boundaries do not map onto locality boundaries which may serve to undermine progress in integration to date.

The benefits of co-location are also very apparent in the Coastal case study. The fact that staff from different sectors and specialties are in the same building facilitates informal conversations, mutual understanding and a willingness to blur professional roles rather than working in service silos. Openness and constant communication help staff to develop a whole team ethos, one in which the risks of doing things differently feel genuinely shared. As a result, leadership in the locality is less ‘transactional’, where top-down management places a strong emphasis on monitoring performance and more ‘transformational’ (i.e. involving participatory and devolved styles) [52] offering greater possibilities for self-organisation and proactive system change.

Because co-location facilitates the development of an informal, engaged and bottom-up dynamic, co-operation, collaboration and integration are more challenging across larger geographical distances. Thus, although integrated care has been identified as a key strategy in rural areas that lack access to specialist services [53,54], it is important to acknowledge that rurality presents both opportunities and barriers to integrated care [55]. Supporting people in their own homes is less cost-effective in sparse settings (such as the Moor to Sea locality) than in the relatively ‘urban’ coastal localities of TSD. The different geographical implications (for staffing, buildings, caseloads, pathways etc) of the national commitment to roll out integrated care deserve greater consideration.

## Strengths and limitations

The longitudinal nature of the data collection is a strength of this study, as is the deployment of embedded Researchers in Residence (RiRs). Managing the expectations of funders and/or stakeholders is a real problem for service evaluators, who are treading a delicate balance between providing ‘evidence’ for highly stressed health service managers who are managing financial pressures and who are acutely aware of their performance targets, while at the same time abiding to an obligation to ‘tell the truth’, which necessitates delving deeply into complexity. The RiR model provides a good solution to this tension.

Using the CCIC Framework helped in the systematic collection, coding and analysis of data and offered the opportunity to compare determinants of implementation success or failure in the English and Canadian contexts. We found similar domains to be crucial, although geography emerged as a more significant factor in this case study. It is important to note that the RiRs spent more time as embedded researchers in the Coastal than other localities, meaning that they gained a more in-depth understanding of this area (a potential limitation). They became part of the contextually situated roles, interactions and relationships under consideration (which might be regarded as either a strength or a limitation, depending on one’s ontological perspective).

## Conclusion

In many health systems, integrated care is seen as the key solution to the growing demand for improved patient experience and health outcomes of patients with complex needs. If integrated care approaches are to be properly adapted to local contexts, a better understanding is required of key determinants of implementation and how these might be appropriately supported. We found that the CCIC Framework provided a useful tool to this end and would encourage its wider application so that further comparisons can be made of the ways in which different contextual and implementation properties impact upon delivery and outcomes.

Our finding that, within one Integrated Care Organisation that is rolling out similar Care Models, variation persists in the processes and outcomes of care coordination suggests that consideration should be given to the *scale* at which interventions are designed, implemented and evaluated. The five localities in TSD face different geographical challenges, comprise different levels of resources (e.g. with respect to staff-mix, the numbers of private care homes, GP vacancies, community assets etc.) and have different histories of collaborative working. These differences matter. However, in an ever-evolving context, they do not present an insurmountable obstacle to replicating the Coastal Locality’s success elsewhere.

Indeed, as members of the Coastal teams have learnt from each other’s experiences, challenges and successes, they are in turn working to learn from and support change within other locality teams and the acute hospital (“where it feels that we are moving from adversarial to collaborative working”), thereby becoming part of an integrated care system that is itself modifying and being modified by key local organisational capabilities (implementation and context becoming inseparable parts of the intervention itself [37]). As in the Canadian study, we found clinical engagement and leadership to be key factors. In the Coastal locality, however, we also highlighted the importance of shared ownership, self-organisation and autonomy. These factors were particularly critical to developing tailored, place-based, population-based approaches that focus on preventing ill health, promoting a shift towards more care in the community and people’s homes and improving person-centred outcomes.
